# Supplementary figures and images for: SARS-CoV-2 sublingual vaccine with RBD antigen and poly(I:C) adjuvant: Preclinical study in cynomolgus macaques
Source: Biol Methods Protoc. 2023 Sep 13;8(1):bpad017. doi: 10.1093/biomethods/bpad017 (PMC10497375; doi:10.1093/biomethods/bpad017)

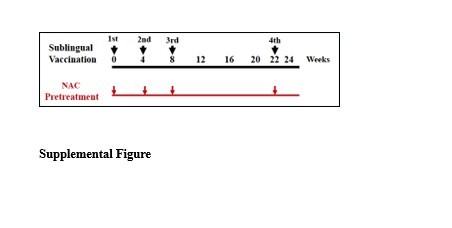

Supplement: bpad017_Supplementary_Data [file bpad017_supplementary_data.jpeg]
